# Indoor particle dispersion due to hand dryer in public washroom: an in silico study

**DOI:** 10.1038/s41598-023-37804-8

**Published:** 2023-07-18

**Authors:** Jing Liao, Zhongjian Ling, Yongou Zhang

**Affiliations:** 1 Tongji Hospital, Tongji Medical College, Huazhong University of Science and Technology, Wuhan, China; 2 School of Naval Architecture, Ocean and Energy Power Engineering, Wuhan University of Technology, Wuhan, China

**Keywords:** Public health, Mechanical engineering

## Abstract

Hand dryer in public washroom has been reported likely to be a reservoir of drug-resistant bacteria. When a hand dryer being used, the high-velocity air jet from the dryer outlet can carry aerosol particles to hand surfaces, the user, and indoor space. This in silico study considered the effect of different airflow speed of hand dryers on the dispersion of particles in different diameters with and without the user. The aim of this study was to apply the computational fluid dynamics (CFD) method based on the discrete phase model to investigate the trajectory of indoor particles from the hand dryer in public washroom. The CFD results showed that, when the user was using the hand dryer, 42.3% of the particles were distributed on the wall against the user, and 31.6% were distributed on the user’s body, including their hands. When no one was standing in front of the hand dryer, 87.6% of the particles fell on the ground. The blocking of user’s hand dispersed the particles to a wide range, particularly for the larger diameter particles which were scattered on the user’s body or on the ground. In addition, the dispersion proportion of particles did not vary with the speed of airflow, but the area of particles distribution became larger as the speed increased. Our findings suggest that the contamination of the indoor environment caused by the hand dryer could not be ignored, incorporating filters into hand dryers is essential. Furthermore, our work offers valuable insights for optimizing the design of hand dryers.

## Introduction

Public washrooms are prone to microorganism growth due to their warm and humid environment. Escherichia coli, Staphylococcus aureus, Shigella spp, and some other pathogens have been shown to survive on various surfaces for days to months^1–3^. These conditions lead to a contaminated environment in the washroom. Contaminated environments can lead to the spread of pathogenic bacteria to the eyes, nose, or mouth of washroom users^4,5^. In addition to toilet facilities and their surroundings^6,7^, contaminated areas also include hand drying facilities. Several studies have shown contamination of hand-drying facilities in public restrooms^8–10^. Effective hand drying reduces cross-contamination compared to dry hand by touching surfaces^11–13^. However, the hand dryer itself can be a source of contamination and disperses microorganism through aerosol particles^14,15^.

Substantial articles have investigated the hygienic effects of hand dryers. Suen et al.^8^ indicated that drug-resistant bacteria could be found in 52 types of bacteria species on hand-drying facilities in public washrooms, and Huesca et al.^9^ demonstrated that hand dryers were related to the bacterial deposition on hand surfaces and the movement of polluted air, and might even be a cause for the dispersion of infectious bacteria. Margas et al.^16^ indicated hand dryers might result in cross contamination of the surrounding environment. Kimmitt and Redway^17^ evaluated the effect of three hand drying methods, namely using jet air dryer, warm air dryer, and paper towel, on the spread of MS2 bacteriophage from contaminated hands, and the jet air dryer showed significantly greater bacterial dispersion than others. Although the link between hand dryers and contaminated environments has been found, the extent and scope of contamination remain unclear, and there is a lack of replication and analysis of the processes that lead to this outcome.

When user uses hand dryer, it will squirt out airflow to quickly evaporate the water on hand surface, which usually lasts a few seconds to a dozen seconds. In the process of air jetting, aerosol particles will disperse to different places in the washroom with the air flow. The influence of factors such as users, airflow speed, particle size, etc. on the particle diffusion process deserves attention. In order to better understand this process, an in silico study is carried out in our work.

Computational fluid dynamics (CFD), as a numerical simulation method, is widely used in environmental forecasting and evaluation^18,19^, and has shown great capability in indoor air flow prediction and quality assessment^20,21^.

In the simulation of environmental particulate pollution, the CFD method has been widely used. On the basis of Navier–Stokes equations, also the main equations for CFD, a momentum source was proposed to simulate the effect of human motion on indoor particles dispersion^22^. With the help of CFD simulation software ANSYS Fluent, Chen et al.^23^ modeled droplet transport and deposition in a simplified mouth-throat model with different boundary conditions, and analyzed the effects of the inlet flow rate and relative humidity. Feng et al.^24^ simulated the transport and deposition of droplets from coughing, and a social distance considering different environmental wind velocities were suggested. In applying the CFD method for the analysis of particles dispersion, the discrete phase model (DPM) method is often used to predict particles trajectories. Chen et al.^25^ used the CFD with DPM method to analyze the deposition of PM2.5 in human airways under rest, light exercise, and moderate exercise conditions. The flow patterns and aerosol trajectories in a typical car, bus, and airplane were simulated with three-dimensional CFD models, and the influence of inflow, outflow, and passenger’s locations were analyzed^26^. CFD simulation of aerosol dispersion inside a grocery store^27^ and a tunnel-ventilated poultry house^28^ based on the DPM method had also been given.

CFD simulation methods also provide excellent assistance in the design optimization of public health devices. The blood flow behavior in a bileaflet mechanical heart valve was investigated based on a finite volume method to improve flow performance^29^. Sommerfeld et al.^30^ reviewed numerical simulations of dry powder inhalers and summed up the role of CFD simulation in the optimization of dry powder inhaler flow channel. The flow velocity and oxygen concentration in the oxygen-supply device installed in a plateau mine were analyzed using the CFD method in the work of Li et al.^31^, and the influence of tunnel airflow velocity was shown. The performance of high flow nasal oxygen devices with a surgical mask on patients was presented by CFD simulations with ANSYS Fluent^32^. The hydraulic performance of the centrifugal blood pump was also optimized by CFD studies^33^. We decided to use this in silico approach to assess particles dispersion due to hand dryers.

In the present study, the process of particle diffusion with airflow from the hand dryer was simulated with an in silico model and the main factors of the particle dispersion were analyzed. A CFD model based on the DPM method was introduced to be a particle tracking approach, and a three-dimensional washroom with hand dryer was built. Furthermore, the influence of airflow velocity, particle diameters and the user on the particle spatial distribution was analyzed, as well as some constructive comments to improve public health are given.

## Methods

### Physical model

**Figure 1:**
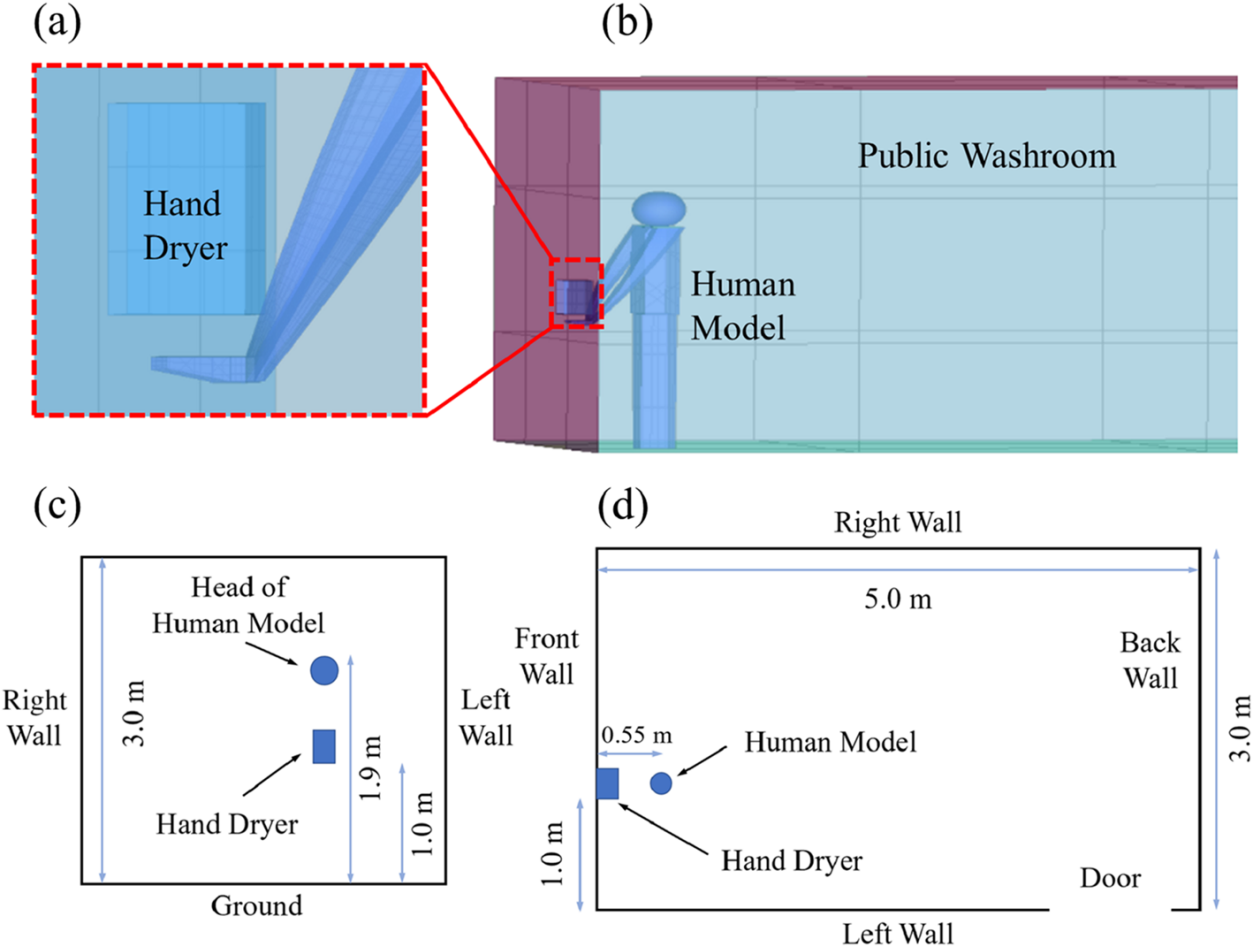
Physical model of the washroom with a hand dryer. (**a**) Enlarged view of the hand dryer and the hand; (**b**) Overall view of the washroom; (**c**) Side view of the washroom; (**d**) Top view of the washroom.

A public washroom model with the length, width and height of 5 m × 3 m × 2.7 m was built. The dimension of the washroom and the position of a hand dryer and a human model are shown in Fig. 1. The size of the hand dryer installed on the washroom wall is 235 mm × 165 mm × 250 mm with 1.0 m away from the ground and 1.0 m away from the left wall. The door of the washroom is 2.1 m × 1.0 m, and it is 0.2 m from the corner. The height of the human model is 1.9 m, and the hand is 50 mm away from the air outlet of the hand dryer. The wall the human model is facing is called the front wall, and the wall to the left/right/back of the human model is called the left/right/back wall respectively.

According to the references, the diameter of the aerosol particles in the air is between 1 μm and 100 μm^32^ , and the density of the aerosol particles is almost the same as that of water^34^. Therefore, in our simulation, the density of the aerosol particle at the outlet of the hand dryer was supposed to be 998.2 kg/m^3^, and the particle diameter was between 1 μm and 100 μm. When the air is ejected from the outlet of the hand dryer, it carries the aerosol particles indoors.

There are usually two types of hand dryers: hot air dryer and jet air dryer. Generally, the airflow speed of the jet air dryer is 15 m per second (MODUN hand dryer, https://www.intelligentvending.co.uk/washroom-accessories/hand-dryers/magnum-standard-1650w-hand-dryer-in-white-abs.htm; Orchids hand dryer for hospitals, https://www.indiamart.com/proddetail/hand-dryer-for-hospitals-10989267862.html. ). Therefore, in the present simulation, cases with different velocities (10 m/s, 15 m/s, 20 m/s) of the airflow from the jet air dryer was modeled.

### Governing equations

#### Airflow in the washroom

The airflow in the washroom was predicted as continuous phase by solving the Navier–Stokes equations including the continuity equation and the momentum equations. After renormalizing the Navier–Stokes equations, the *Re*-Normalization Group (RNG) k-ε model was adopted to predict the turbulent flow. The corresponding governing equations in tensor notation are given as:

1 $$\frac{\partial (\rho k)}{\partial t}+\frac{\partial (\rho k{u}_{i})}{\partial {x}_{i}}=\frac{\partial }{\partial {x}_{j}}\left[\left(\mu +\frac{{\mu }_{t}}{{\sigma }_{k}}\right)\frac{\partial k}{\partial {x}_{j}}\right]+{P}_{k}-\rho \varepsilon$$

2 $$\frac{\partial (\rho \varepsilon )}{\partial t}+\frac{\partial (\rho \varepsilon {u}_{i})}{\partial {x}_{i}}=\frac{\partial }{\partial {x}_{j}}\left[\left(\mu +\frac{{\mu }_{t}}{{\sigma }_{\varepsilon }}\right)\frac{\partial \varepsilon }{\partial {x}_{j}}\right]+{G}_{1\varepsilon }\frac{\varepsilon }{k}{P}_{k}-{G}_{2\varepsilon }^{*}\rho \frac{{\varepsilon }^{2}}{k}$$

where *ρ* is the air density, *k* is the turbulent kinetic energy, ε is the dissipation rate, *t* is time, *u*_*i*_ (i = 1, 2, 3) is the air velocity in *x, y, z* directions, *x*_*i*_ and *x*_*j*_ (i = 1, 2, 3) are space in *x, y, z* directions, *μ* is the dynamic viscosity, *σ*_*k*_ and *σ*_*ε*_ are the turbulent Prandtl numbers, and *P*_*k*_ is the turbulent kinetic energy due to the mean velocity gradients. *C*_*1ε*_, *C*_*2ε*_, *σ*_*k*_, and *σ*_*ε*_ are model constants, and their values are 1.44, 1.92, 1.0, and 1.3 respectively. *μ*_t_ is the turbulent dynamic viscosity defined by:

3 $${\upmu }_{t}=\rho {C}_{\upmu }\frac{{k}^{2}}{\varepsilon }$$

where the model constant ${C}_{\upmu }$ is 0.09.

Our work mainly considered the dispersion of particles in steady air flow, so the variation of variables with time was ignored in the solution.

#### Particle movement

Aerosols carried in jets from the hand dryer are supposed to be small particles, which is a conventional assumption^24,26^, and the particles are usually simulated by the DPM method. Ignoring the rotation of the particle itself, the particle trajectory can be obtained according to the Newton's second law, i.e.,

4 $$\frac{d{u}_{pi}}{dt}={F}_{i}^{D}\left({u}_{i}-{u}_{pi}\right)+\frac{{g}_{i}\left({\rho }_{p}-\rho \right)}{{\rho }_{p}}+{F}_{i}$$

where *u*_*pi*_ is the particle velocity, *g*_*i*_ is gravity, *ρ*_*p*_ is the density of particle, and *F*_*i*_ is the additional force per unit mass. *F*_*i*_^*D*^ (*u*_*i*_*-u*_*pi*_ ) is the drag force per unit particle mass and *F*_*i*_^*D*^ can be given by:

5 $${F}_{i}^{D}=\frac{18\mu }{{\rho }_{p}{d}_{p}^{2}}\frac{{C}_{D}{Re}_{d}}{24}$$

where *d*_*p*_ is the particle diameter, *R*_*ed*_ is the Reynolds number defined on the particle diameter, and *C*_*D*_ is the drag force coefficient defined as:

6 $${\mathrm{C}}_{\mathrm{D}}={\mathrm{a}}_{1}+\frac{{\mathrm{a}}_{2}}{{\mathrm{Re}}_{\mathrm{d}}}+\frac{{\mathrm{a}}_{3}}{{\mathrm{Re}}_{\mathrm{d}}^{2}}$$

where *a*_*1*_, *a*_*2*_, and *a*_*3*_ are model constants.

### Computational model and boundary conditions

**Figure 2:**
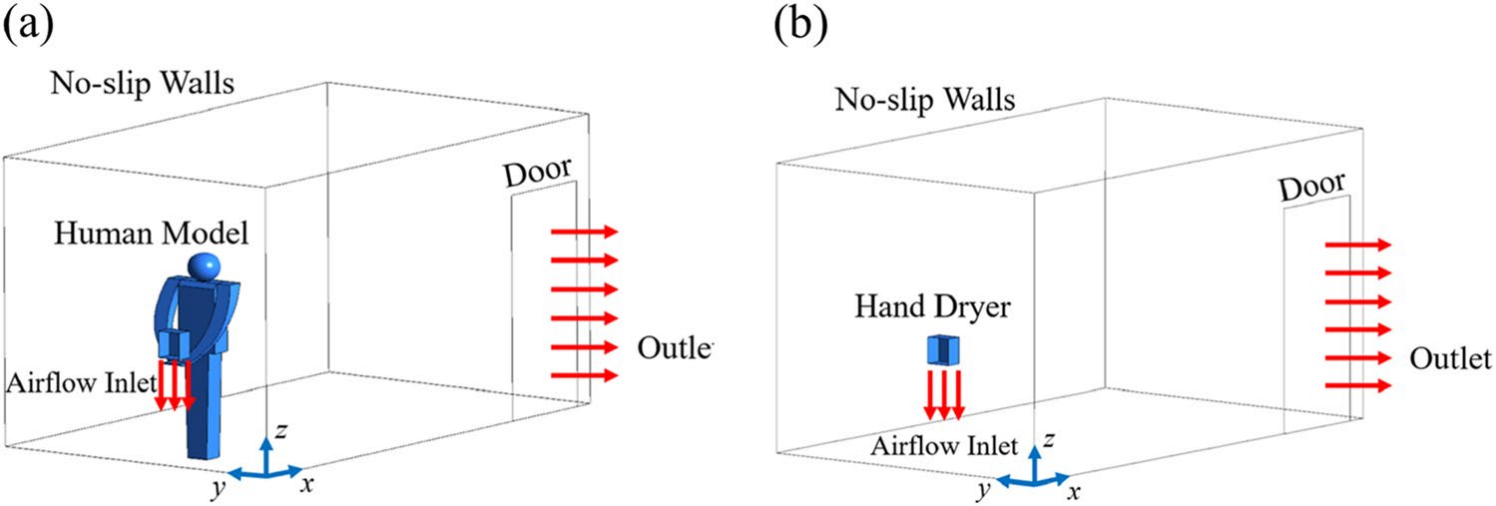
Boundary conditions of the CFD model. (**a**) with human model, corresponding to the situation when someone is using the hand dryer; (**b**) without human model, corresponding to the situation that the user has just left.

A commercial software, ANSYS Fluent, (ANSYS, U.S.A) as an industry-leading fluid simulation software is known for its advanced physics modeling capabilities and industry leading accuracy, and is applied to solve the governing equations for the airflow and predict the particle dispersion. In the simulation, flow in the washroom was considered as steady-state flow and solved before simulating the particle motion. The boundary conditions are shown in Fig. 2. A coordinate system is also given in the figure for the convenience of data description. The inlet flow from the hand dryer is defined as the velocity boundary condition, and the door is defined as the pressure outlet. All walls, the ground and the hand dryer case are defined as no-slip walls. Ten thousand particles are released from the inlet. Once the particles hit the walls, they stick to the wall and stop moving. Both someone and no one using the hand dryer in the washroom were considered in our work.

**Figure 3:**
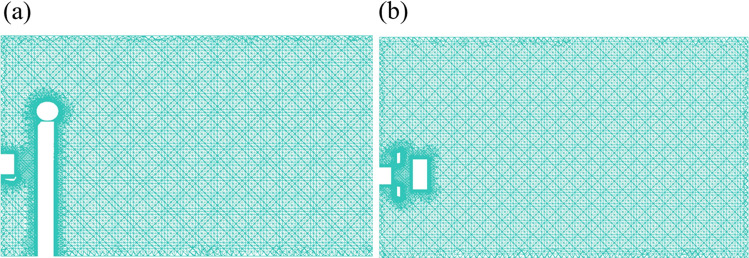
CFD mesh of the washroom with the human model. (**a**) front view; (**b**) top view.

In order to understand the movement of particles, the washroom is spatially discretized based Coupled method, and a CFD mesh is obtained by the software ICEM CFD as shown in Fig. 3, which is an unstructured mesh. Mesh independence tests had been performed for the inlet velocity at 15 m/s. The DPM method and simulation software ANSYS Fluent have been well validated in previous studies^26,29,32^.

## Results and discussion

### Human model effect on particle dispersion

**Figure 4:**
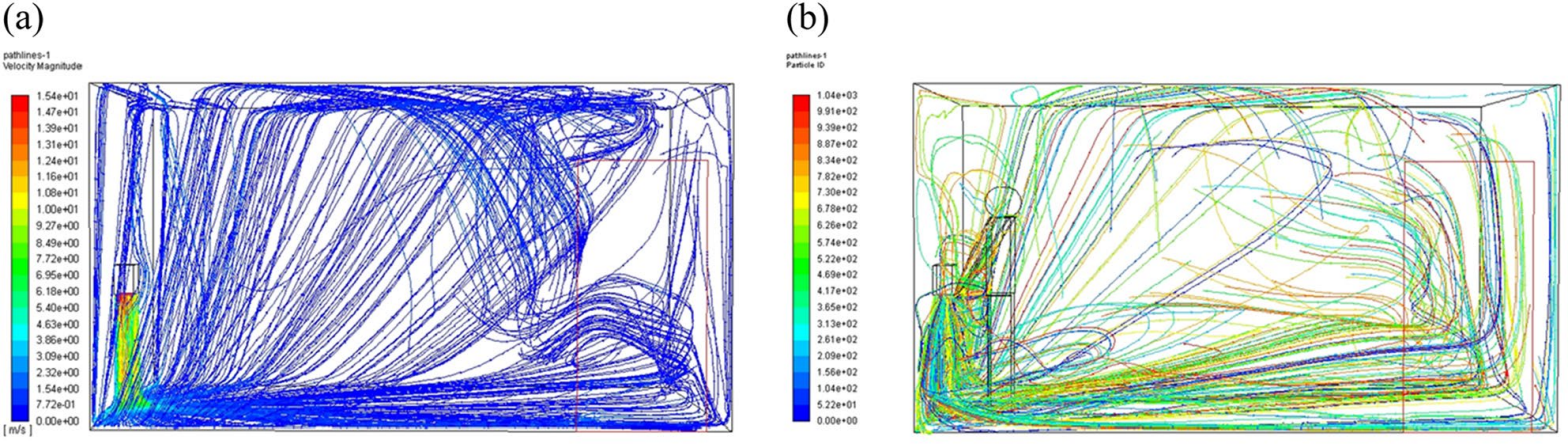
Velocity vector diagram in the washroom at end time. (**a**) Velocity vector, no human; (**b**) Velocity vector, one human.

**Figure 5:**
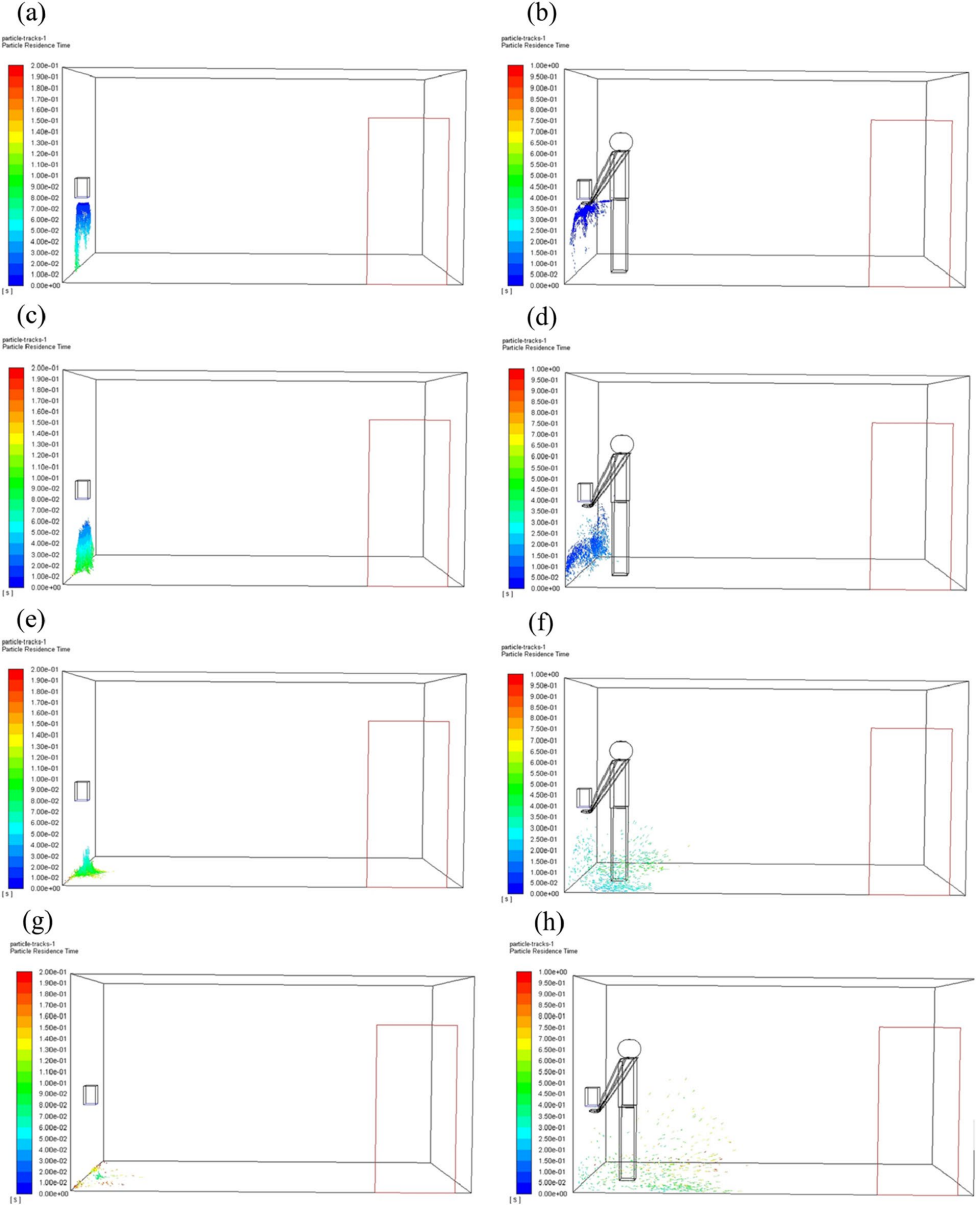
Distribution of particles in the washroom at different time. It should be noted that once the particles attached to the wall or the human body, they were no longer displayed in the figure. (**a**, **c**, e, g) Hand dryer is working with no human; (b, d, **f**, **h**) Hand dryer is working with a user in front.

When the hand dryer is working, particles are moving with airflow from the outlet of the hand dryer and disperse to the room. The velocity vector diagram in the washroom is shown in Fig. 4. The distribution of particles in space at different time are shown in Fig. 5, and they are all instantaneous snapshots. The airflow speed of the hand dryer was 15 m/s and the particle diameter was 50 μm. The particle distribution with no user in front of the hand dryer (Fig. 5a, c, e, g) show that the particles mainly fall to the ground right below the hand dryer. When someone was using the hand dryer, the particles spread around due to the blocking effect of the hand (Fig. 5b, d, f, h).

**Figure 6:**
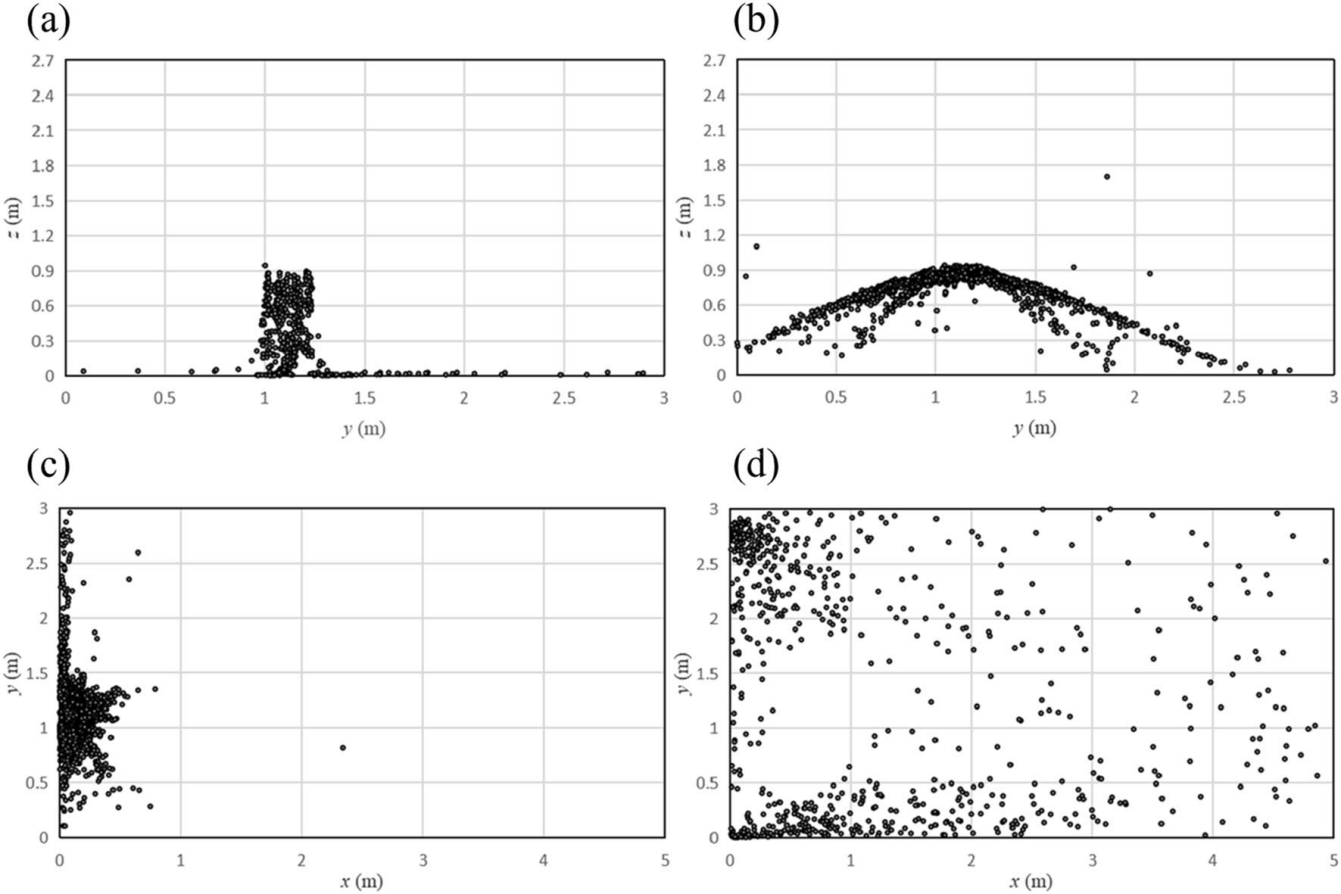
Distribution of particles on different walls. (**a**) Front wall, no human; (**b**) Front wall, one human; (**c**) Ground, no human; (**d**) Ground, one human.

After releasing 10 thousand particles, the distribution of particles on the front wall and the ground is shown in Fig. 6. Figure 6a show that, when no one was in front of the hand dryer, the particles fell along the direction of the jet wind and gathered directly under the hand dryer. From Fig. 6b, it can be seen that the particles were blown away in all directions instead of gathered under the hand dryer because of the blocking effect of the hand. Similar results can also be seen from the particle distribution on the ground. The particles in Fig. 6c) gathered on the ground directly under the hand dryer, and the particles in Fig. 6 (d) gathered in the corner of the public washroom.

As can be seen from the above results, if someone is using a hand dryer, it becomes difficult to confirm the direction in which the particles spread and where they end up. For the physical model in our work, the particles gather in the corners of the room, but if you change to a different room, or consider the changing position of the user's hand, the distribution of the particles may be different. As people use and leave (while the hand dryer continue to work for a while), there is no doubt that the particles will disperse to a large area below the hand dryer.

**Figure 7:**
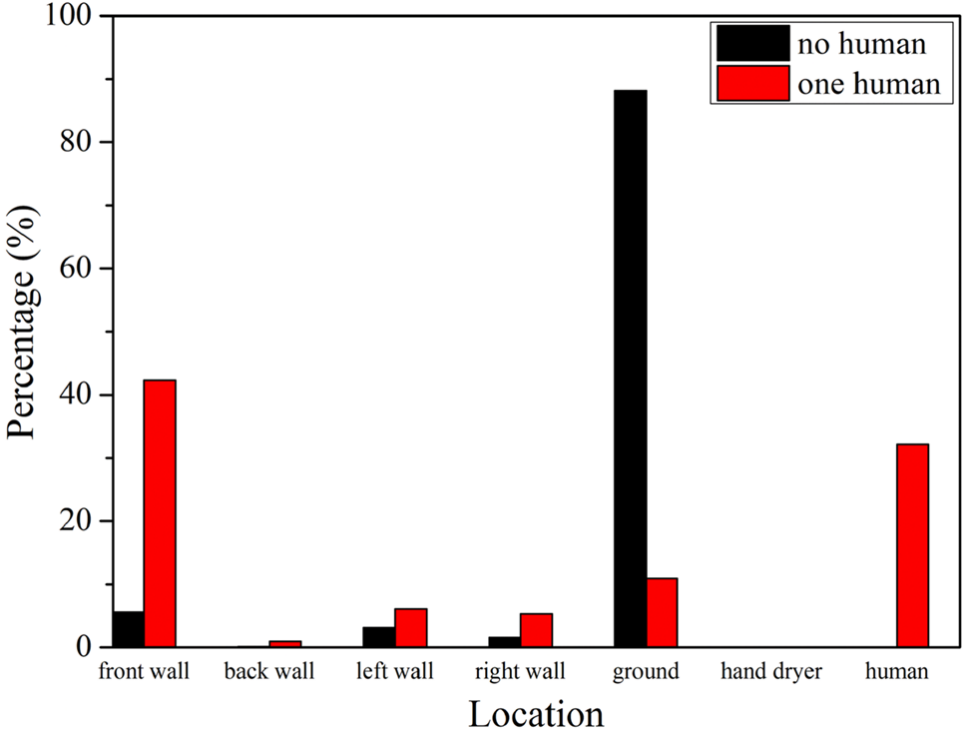
Percentage of particles on different walls and the human model with airflow speed at 15 m/s.

The percentage of particle distribution on different walls and the human model with airflow speed at 15 m/s is shown in Fig. 7. When no one was in front of the hand dryer, the number of particles falling on the ground account for 88.2% of the total number of particles, which was much more than other walls. When someone was using the hand dryer, the percentage of particles sticking to the front wall reached 42.3%, followed by the human model which was 32.2%. At the same time, the percentage of particles on the ground was around 11.0%.

It is clear that the distribution of particles is completely different when someone is using the hand dryer and when no one is using the hand dryer. The reason of such a different is the blocking effect of the hand, which makes the particle movement very different. The hand causes the particles to spread out on a horizontal plane, rather than rushing to the ground with gravity and airflow. Since the front wall and the human body are close to the hand, a large number of particles fall on them. Thus it is essential to add filters when designing the hand dryers.

### Airflow velocity effect on particle dispersion

**Figure 8:**
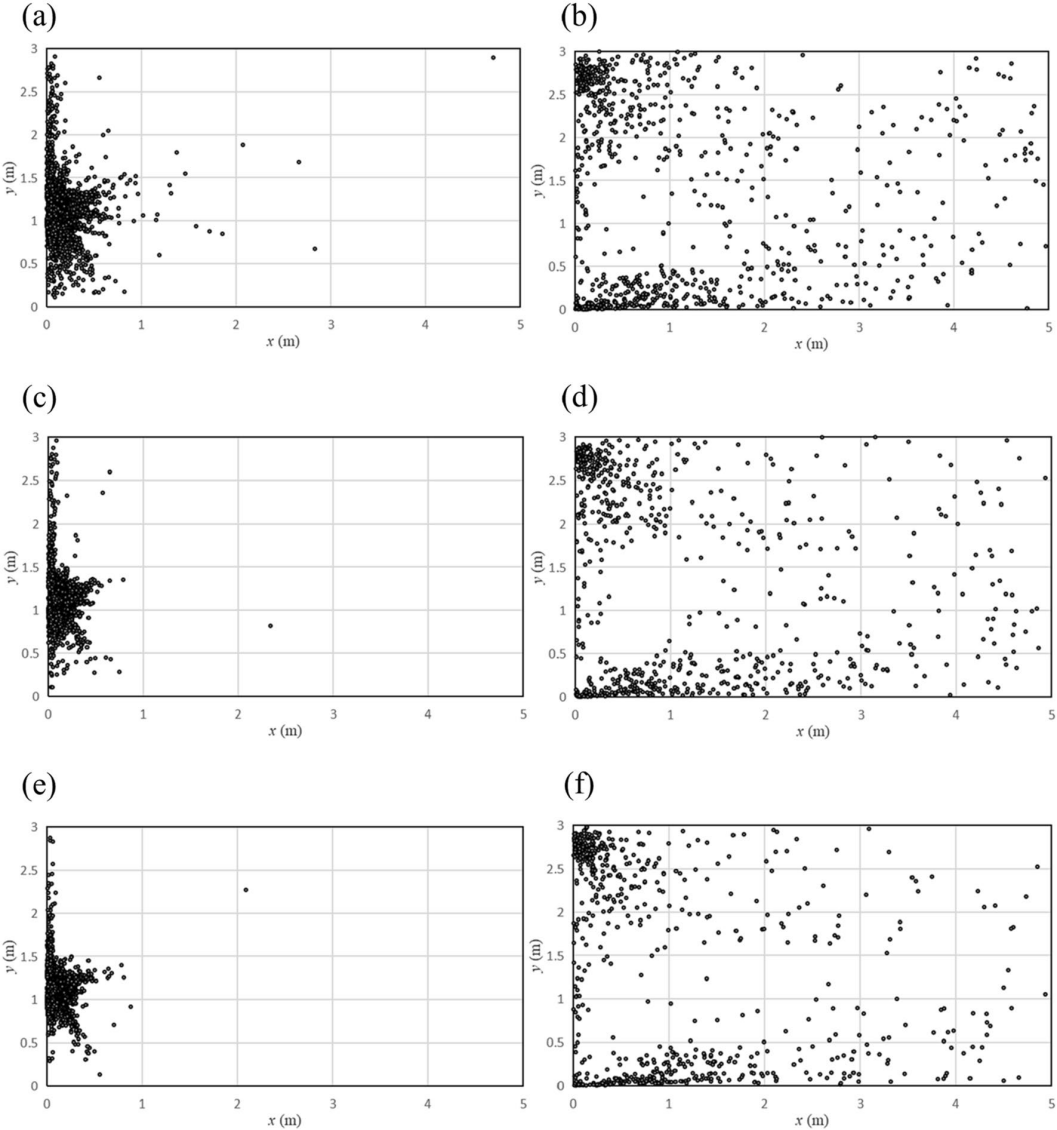
Distribution of particles on the ground with different airflow speed. (**a**) No human, 10 m/s; (**b**) One human, 10 m/s; (**c**) No human, 15 m/s; (**d**) One human, 15 m/s; (**e**) No human, 20 m/s; (**f**) One human, 20 m/s.

The distribution of particles on the ground under different airflow speed is shown in Fig. 8, where the left column shows the situation when there were no people in the washroom, and the right column show the situation when there was a user. As the flow rate increases, more and more particles gathered.

When no one was in front of the hand dryer, as the airflow velocity increased, the particles on the ground got closer and closer to the wall, i.e. directly below the hand dryer. When someone was using the hand dryer, as the airflow velocity increased, the particle density in the corners of the room increased and the distribution of particles became more concentrated.

Although the location of particles changed with increasing airflow velocity, the percentage of particles on the walls and the human model did not change much. Percentage of particles at different locations with different airflow speed are listed in Table 1. Since the percentage of particles distributed on each wall did not change much with the flow velocity, we only give the percentage of particles distributed on three walls. When no one was in the washroom, the percentage of particles falling on the ground remained at around 88%. When someone was using the washroom, the percentage of particles falling on the front wall and the human model remained at about 42% and 32% respectively.

**Table 1:** Percentage of particles at different locations at different airflow speed.

Title 1	10 m/s (%)	15 m/s (%)	20 m/s (%)
Ground (no human)	87.64	88.16	88.53
Front wall (one human)	42.26	42.31	42.52
Human (one human)	31.64	32.17	32.57

### Particle diameter effect on particle dispersion

**Figure 9:**
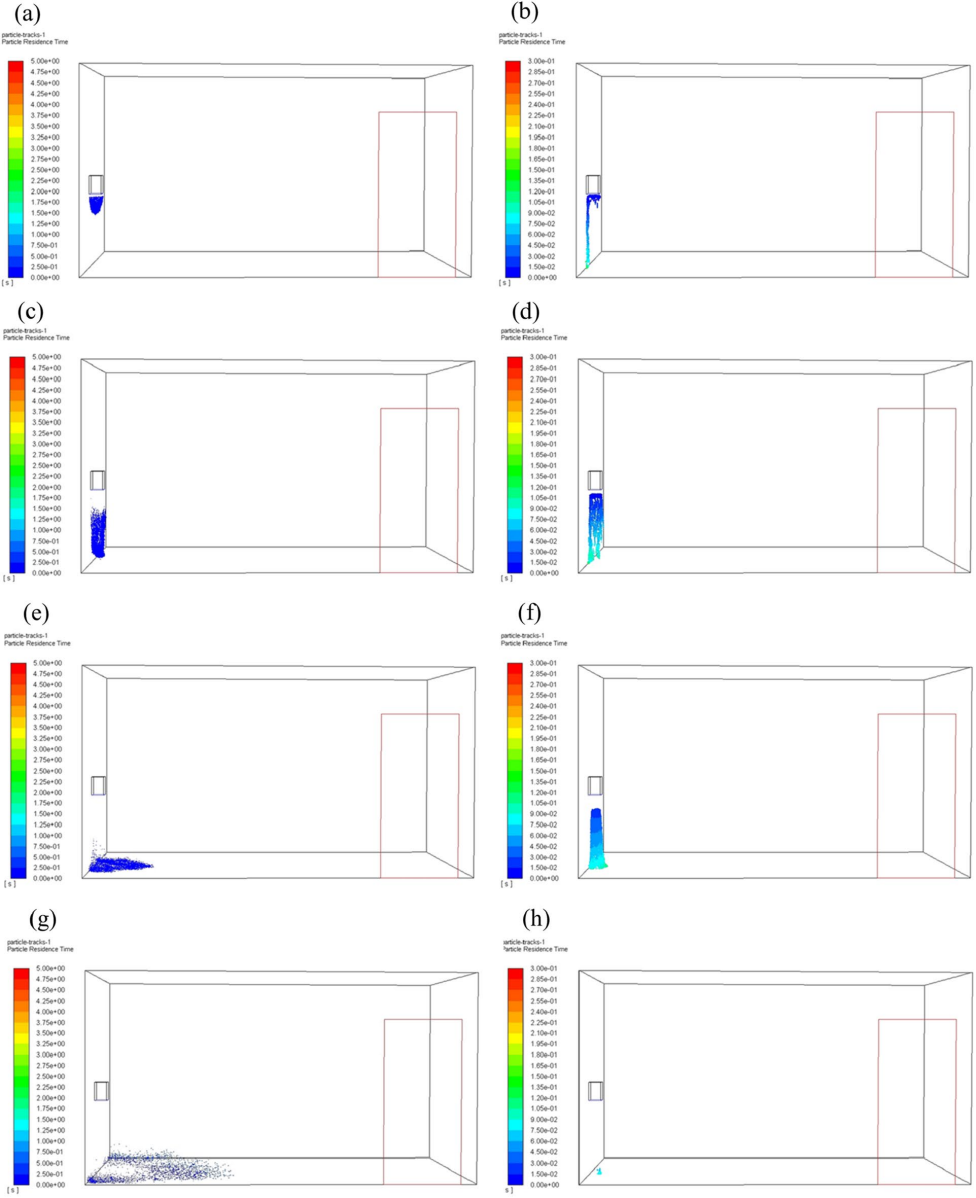
Distribution of particles with different diameters floating in the air at different time when no one is in front of the hand dryer. (**a**, **c**, **e**, **g**) Particle diameter 1 μm; (**b**, **d**, **f**, **h**) Particle diameter 100 μm.

**Figure 10:**
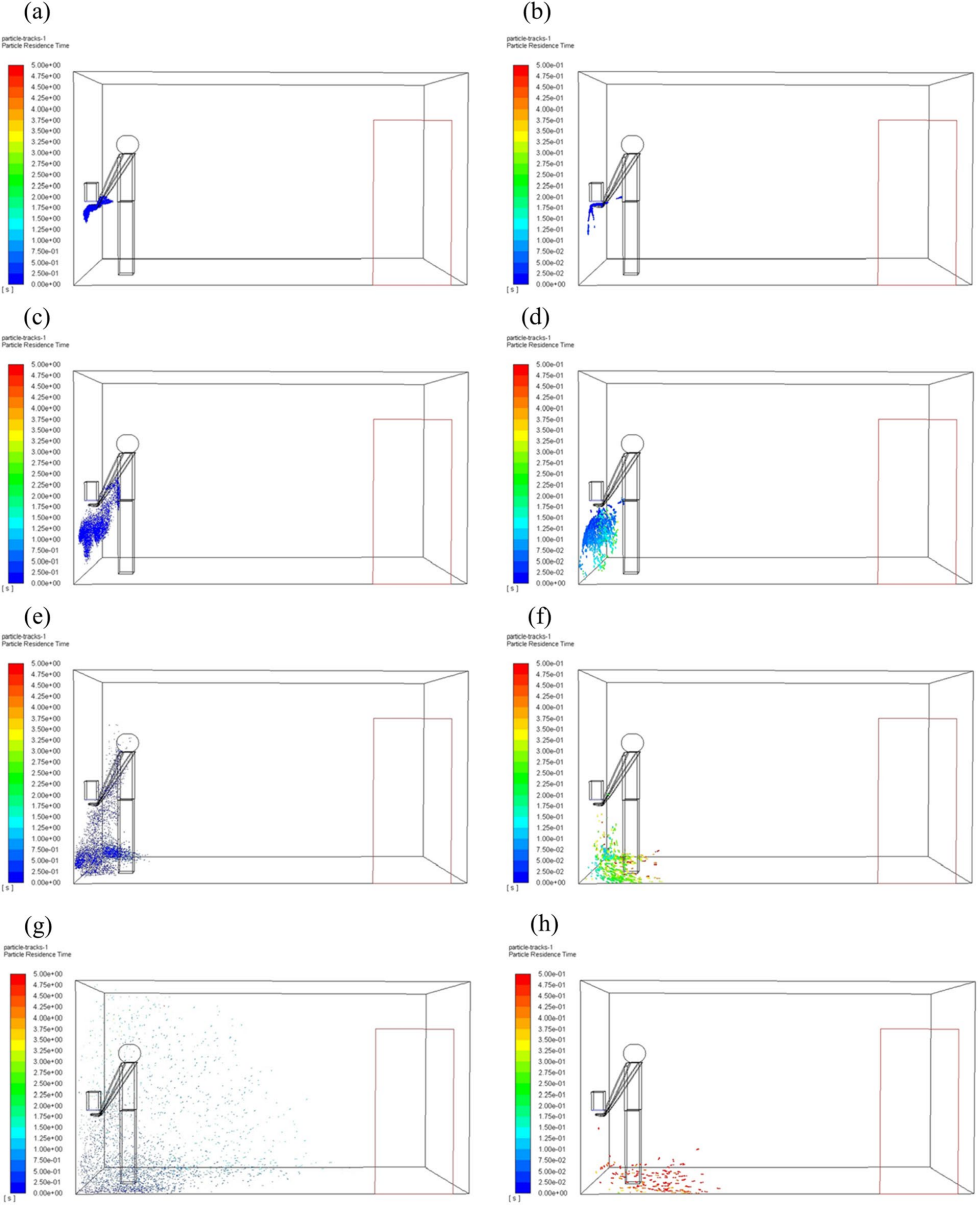
Distribution of particles with different diameters floating in the air at different time when the user is in front of the hand dryer. (**a**, **c**, **e**, **g**) Particle diameter 1 μm; (**b**, **d**, **f**, **h**) Particle diameter 100 μm.

In our simulation, the particles carried by the air jet from the hand dryer contained three different sizes, but the difference in particle size was not analyzed in previous sections. In this section, we analyzed the effect of particle diameter on particle dispersion when the airflow speed was at 15 m/s. Particles with different diameters floating in the air at different time are shown in Fig. 9 and Fig. 10 (they are all instantaneous snapshots), where the left column corresponds to the particle diameter of 1 μm, and the right column corresponds to the particle diameter of 100 μm. It should be noted that once the particles attach to the wall or the human body, they are no longer displayed in the figure.

After people leave the hand dryer, the particles with large diameter and weight quickly fall to the ground. On the contrary, the particles with small size and light weight disperse to a larger area near the ground. When someone is using the hand dryer, the particles spread over a wide area, and the particles with large diameter still fall to the ground faster and more aggregated than smaller particles.

**Figure 11:**
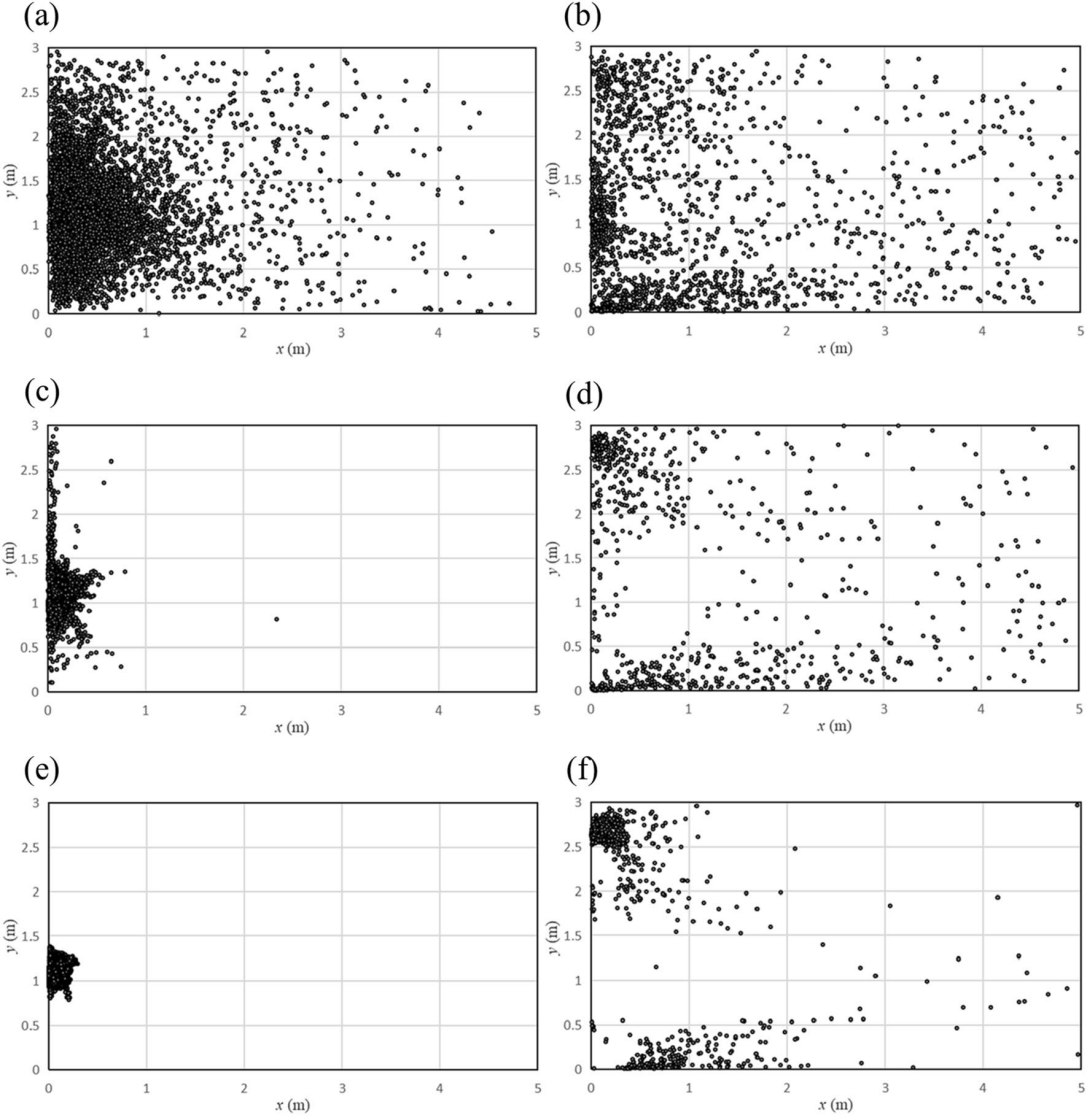
Distribution of particles on the ground with different particle diameters. (**a**) No human, 1 μm; (**b**) One human, 1 μm; (**c**) No human, 50 μm; (**d**) One human, 50 μm; (**e**) No human, 100 μm; (**f**) One human, 100 μm.

Distribution of particles falling on the ground with different particle diameters is shown in Fig. 11. It can be seen from the figure that the diffusion range of particles with a smaller diameter is significantly larger than that of particles with a larger diameter. When there was no one in front of the hand dryer, most of the ground (x > 1 m) was free of particles with the diameter of 50 μm and 100 μm, and the particles with the diameter of 1 μm spread all over the ground. A similar trend can be seen when there was a user in front of the hand dryer, and the particle aggregation occurred in the corner of the room.

**Figure 12:**
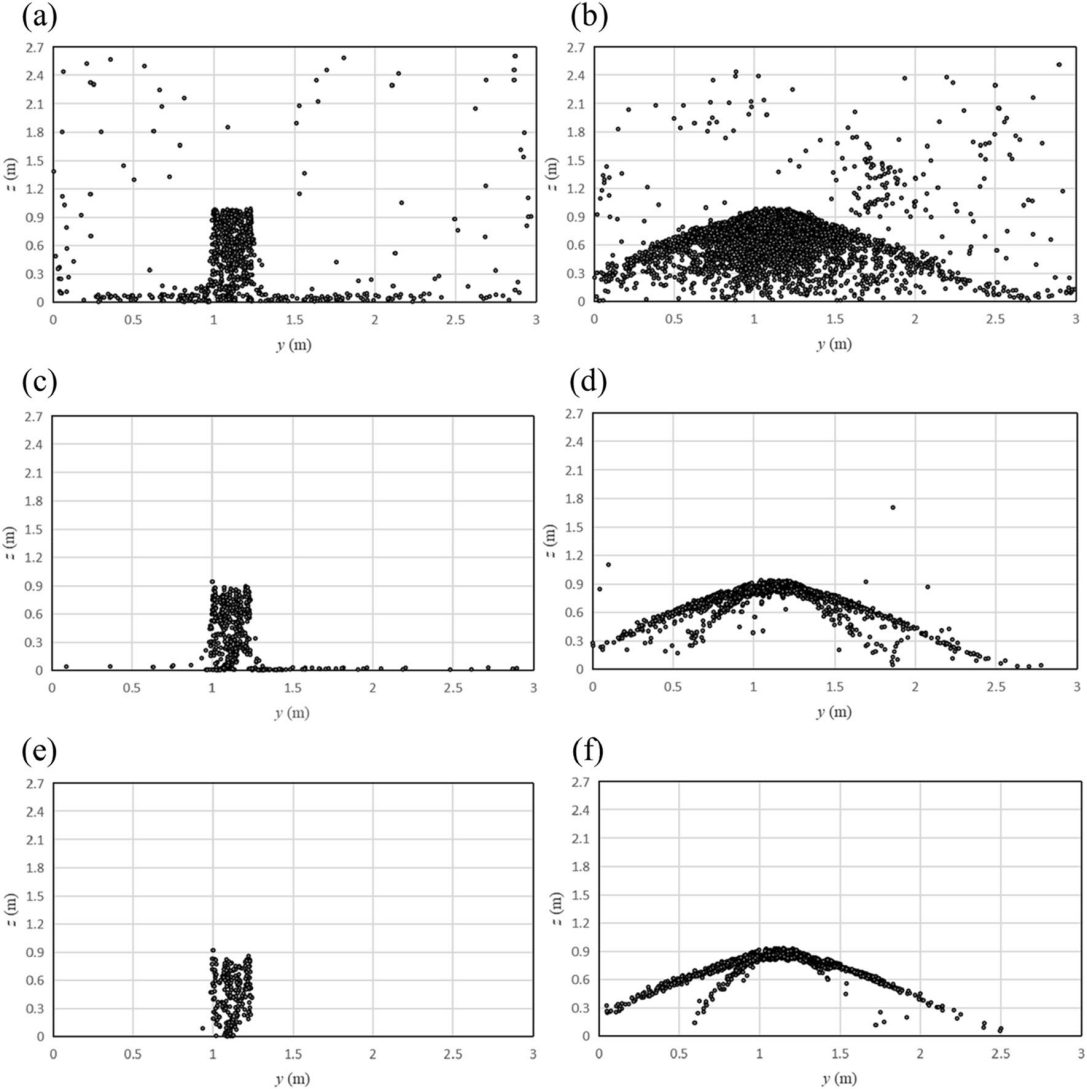
Distribution of particles on the front wall with different particle diameters. (**a**) No human, 1 μm; (**b**) One human, 1 μm; (**c**) No human, 50 μm; (**d**) One human, 50 μm; (**e**) No human, 100 μm; (**f**) One human, 100 μm.

Considering the number of particles on the front wall exceeds 40% when someone is using the hand dryer, Fig. 12 show the distribution of particles on the front wall with different particle diameters. It can be seen that the larger the particle diameter, the more concentrated the area of particle distribution. When the particle diameter was 1 μm, the particles were visible in most area of the wall, whether or not anyone is using the hand dryer. When the particle diameter was over 50 μm, the particles fell directly under the hand dryer or splash in four directions due to the blocking of the human hand.

**Figure 13:**
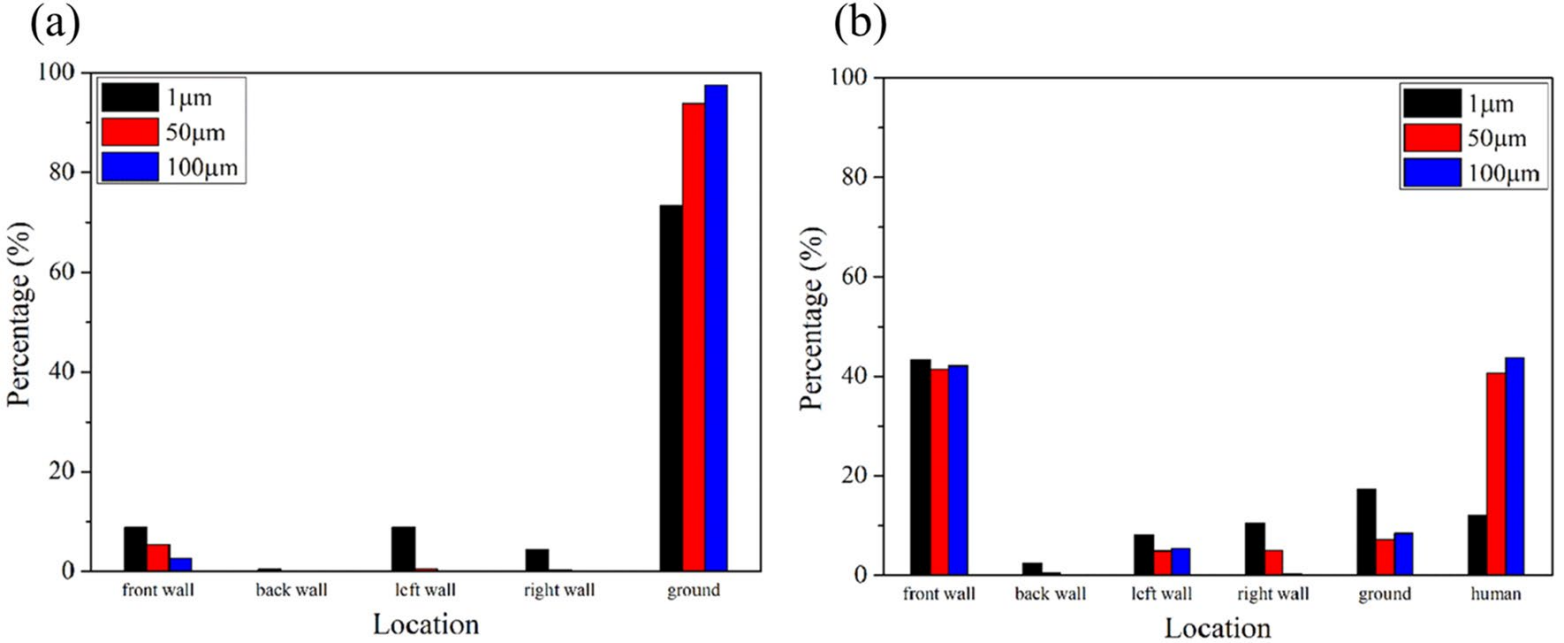
Percentage of particles on different walls and the human model with different particle diameters. (**a**) No one in front of the hand dryer; (**b**) One user in front of the hand dryer.

Percentage of particles with different diameters on walls and the human model are shown in Fig. 13. When no one was in front of the hand dryer, as the diameter increased, so does the number of particles that fell on the ground. When the particle diameter was 100 μm, the percentage of particles that fell on the ground reach a maximum value of 97.5%.

When someone is using the hand dryer, the number of particles that fall on the human body, including the hand, are significantly influenced by the particle diameter. The larger the particle diameter, the greater the number of particles that fall on the human model. When the particle diameter is 100 μm, the percentage of particles that fall on the human model reaches a maximum value of 43.7%. On the contrary, the particle size has little effect on the number of particles on the wall facing the user.

## Conclusions

The hand dryer in the public washroom has been reported likely to be a reservoir of drug-resistant bacteria. The CFD simulation was performed to analyze the dispersion of particles moving with the airflow from the hand dryer. The effects of human model, airflow speed, and the particle diameter on the particle distributions were explored, which provides a reference for the optimal design of hand dryers. Several results are summarized as follows:

(i) When someone is using the hand dryer, the particles moving with the airflow from the hand dryer will fall into the corner of the room. The percentage of particles on the front wall and the human model are 42.3% and 32.2% respectively. When the user leaves, if the hand dryer is still running, most of the particles fall on the ground directly below the hand dryer and the number of particles on the ground is 88.2% of the total number of particles.
(ii) The airflow speed of the hand dryer, from 10 m/s to 20 m/s, has little effect on the percentage of particles distributed on the walls and the human body.
(iii) The smaller the diameter of the particles, the wider the dispersion range, and the larger the diameter of the particles, the easier it is to fall directly on the ground or the human hand.

## Limitations and further work

The numerical model in this paper may be different from the actual situation. We have not found a similar simulation analysis of the problems discussed in this paper in previous research literatures. More accurate and more realistic research results need to be further improved.

## Data Availability

The datasets used and/or analysed during the current study available from the corresponding author on reasonable request.
